# Supplementary material for: In vivo rat brain mapping of multiple gray matter water populations using nonparametric D(ω)‐R 1‐R 2 distributions MRI
Source: NMR Biomed. 2024 Nov 24;38(1):e5286. doi: 10.1002/nbm.5286 (PMC11628177; doi:10.1002/nbm.5286)
Supplement: Supplementary file 1 — Figure S1: Parameter maps derived from the per‐voxel D (ω)‐R 1‐R 2‐distributions of the in vivo rat brain slice 5. (a) Non‐weighted signal S 0 = S(b=0, 𝜏R → ∞, 𝜏E = 0) obtained by Equation 7; graphical representation of the 1/2/3 bins in the 2D D iso‐D Δ2 space calculated according to the bin limit defined in the multidimensional diffusion data inversion section; and bin‐resolved signal fractions f bin1/bin2/bin3 coded into RGB color. The bin limits are selected to resolve white matter (WM), gray matter (GM), and cerebrospinal fluid (CSF). Primary colors indicate voxels containing pure WM, GM, or CSF while mixed colors show voxels with partial volumes of WM + GM (yellow), WM + CSF (purple), or GM + CSF (turquoise). (b) Per‐voxel means E[x], variances V[x], and covariances C[x,y] of the D iso, D Δ2, R 1, and R 2 dimensions. (c) Per‐voxel rates of change with frequency (Δω/2π) means Δω/2πE[x], variances Δω/2πV[x], and covariances Δω/2πC[x,y] of the D iso, D Δ2 dimensions. The ω‐dependent parameters (D iso and D Δ) were calculated at the low sampling frequency of ω cent = 18 Hz and their corresponding Δω/2π values between ω cent = 18 and 92 Hz. (d) Bin‐resolved signal fractions f bin1/bin2/bin3, means (E[x] and Δω/2πE[x]) coded into image brightness and color, respectively. The direction‐encoded color is based on the lab‐frame diagonal values D xx, D yy, and D zz normalized by the maximum eigenvalue D 33. Figure S2: Parameter maps derived from the per‐voxel D(ω)‐R1‐R2‐distributions of the in vivo rat brain slice 3. Figure S3: Parameter maps derived from the per‐voxel D(ω)‐R1‐R2‐distributions of the in vivo rat brain slice 2. Figure S4: Parameter maps derived from the per‐voxel D(ω)‐R1‐R2‐distributions of the in vivo rat brain slice 1. Figure S5: Histograms of quantitative parameters median values across all voxels in the image. The histograms are obtained from the per‐voxel (gray) and bin‐resolved (red, green, and blue) maps of E[Diso], E[DΔ2], E[R1], E[R2], Δω/2πE[Dis [file NBM-38-e5286-s001.docx]

**Supplementary information:**

***In vivo* rat-brain mapping of multiple gray matter water populations using nonparametric D(*ω*)-*R*1-*R*2 distributions MRI**

Maxime Yon^1,2^, Omar Narvaez^1^, Daniel Topgaard^2^, and Alejandra Sierra^1^

^1^A.I. Virtanen Institute for Molecular Sciences, University of Eastern Finland, Kuopio, Finland.

^2^Department of Chemistry, Lund University, Lund, Sweden.


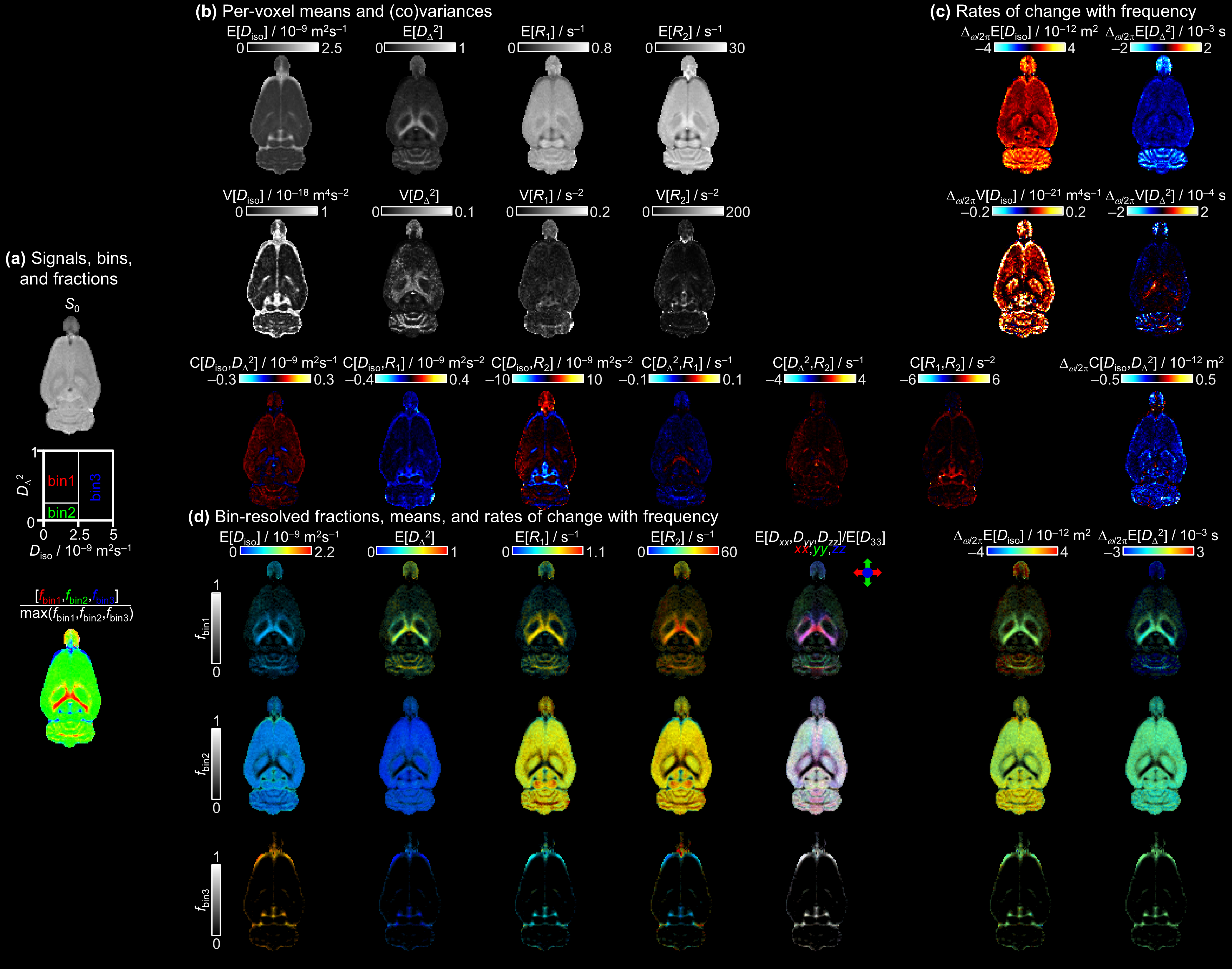


Supplementary Figure 1:  Parameter maps derived from the per-voxel ***D***(*ω*)-*R*_1_-*R*_2_-distributions of the in vivo rat brain slice 5. (a) Non-weighted signal *S*_0_ = *S*(*b*=0, 𝜏_R_ → ∞, 𝜏_E_ = 0) obtained by Eq. 7; graphical representation of the 1/2/3 bins in the 2D *D*_iso_-*D*_Δ_^2^ space calculated according to the bin limit defined in the multidimensional diffusion data inversion section; and bin-resolved signal fractions *f*_bin1/bin2/bin3_ coded into RGB color. The bin limits are selected to resolve white matter (WM), gray matter (GM), and cerebrospinal ﬂuid (CSF). Primary colors indicate voxels containing pure WM, GM, or CSF while mixed colors show voxels with partial volumes of WM + GM (yellow), WM + CSF (purple), or GM + CSF (turquoise). (b) Per-voxel means E[x], variances V[x], and covariances C[x,y] of the *D*_iso_, *D*_Δ_^2^, *R*_1_, and *R*_2_ dimensions. (c) Per-voxel rates of change with frequency (Δ_ω/2π_) means Δ_ω/2π_E[x], variances Δ_ω/2π_V[x], and covariances Δ_ω/2π_C[x,y] of the *D*_iso_, *D*_Δ_^2^ dimensions. The ω-dependent parameters (*D*_iso_ and *D*_Δ_^2^) were calculated at the low sampling frequency of *ω*_cent_ = 18 Hz and their corresponding Δ_ω/2π_ values between *ω*_cent_ = 18 and 92 Hz. (d) Bin-resolved signal fractions *f*_bin1/bin2/bin3_, means (E[x] and Δ_ω/2π_E[x]) coded into image brightness and color, respectively. The direction-encoded color is based on the lab-frame diagonal values *D*_xx_, *D*_yy_, and *D*_zz_ normalized by the maximum eigenvalue *D*_33_.


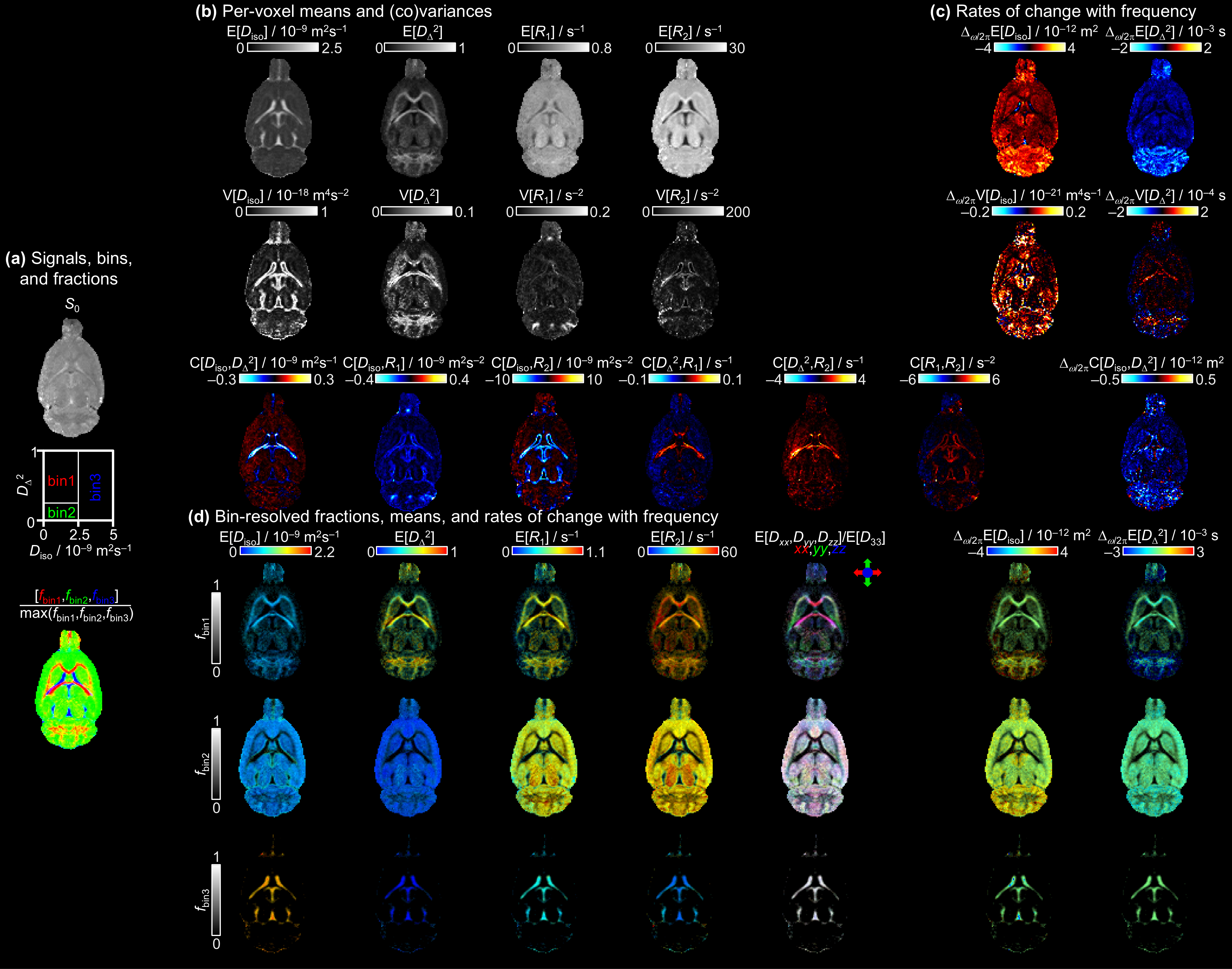


Supplementary Figure 2: Parameter maps derived from the per-voxel **D**(ω)-R1-R2-distributions of the in vivo rat brain slice 3.


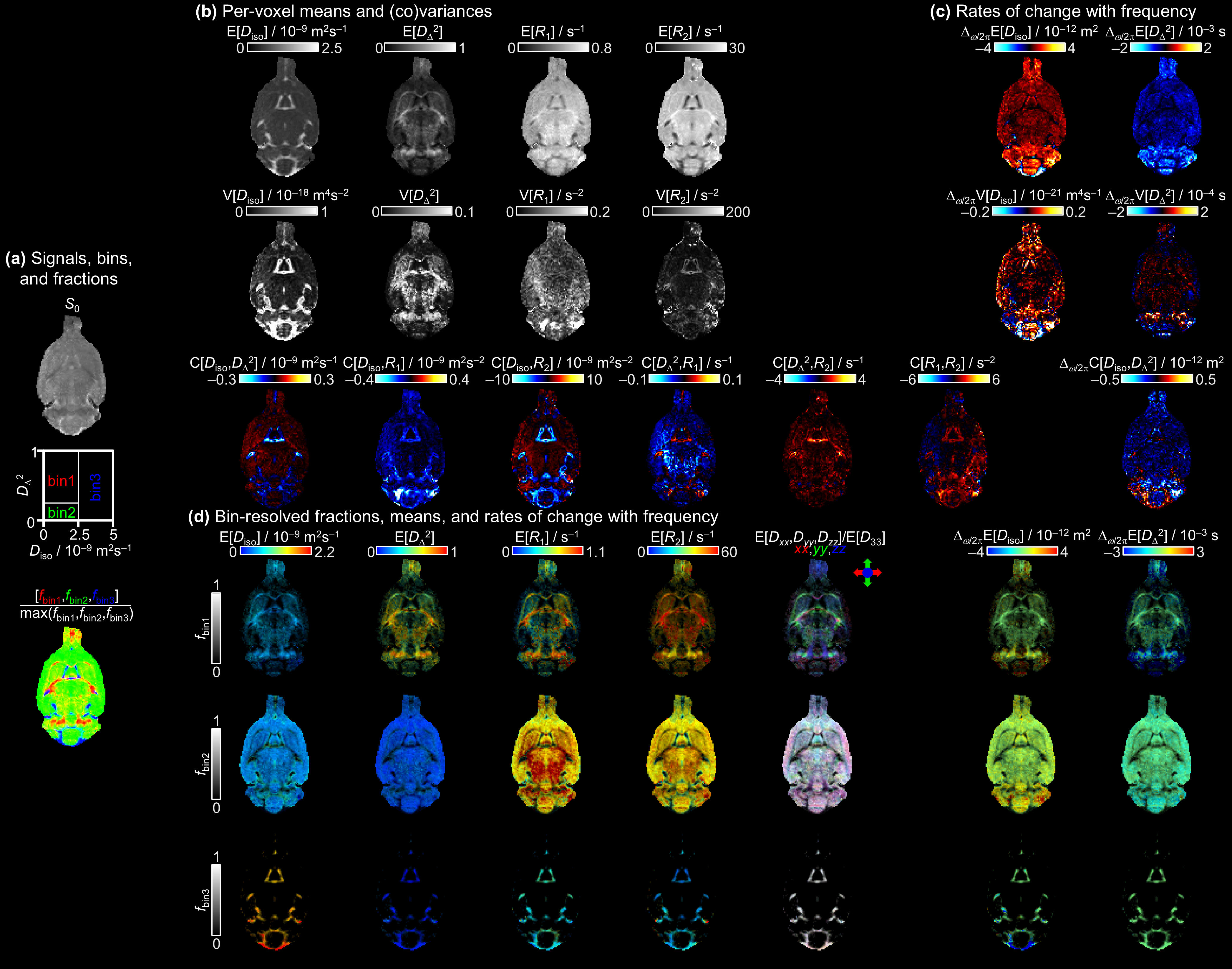


Supplementary Figure 3: Parameter maps derived from the per-voxel **D**(ω)-R1-R2-distributions of the in vivo rat brain slice 2.


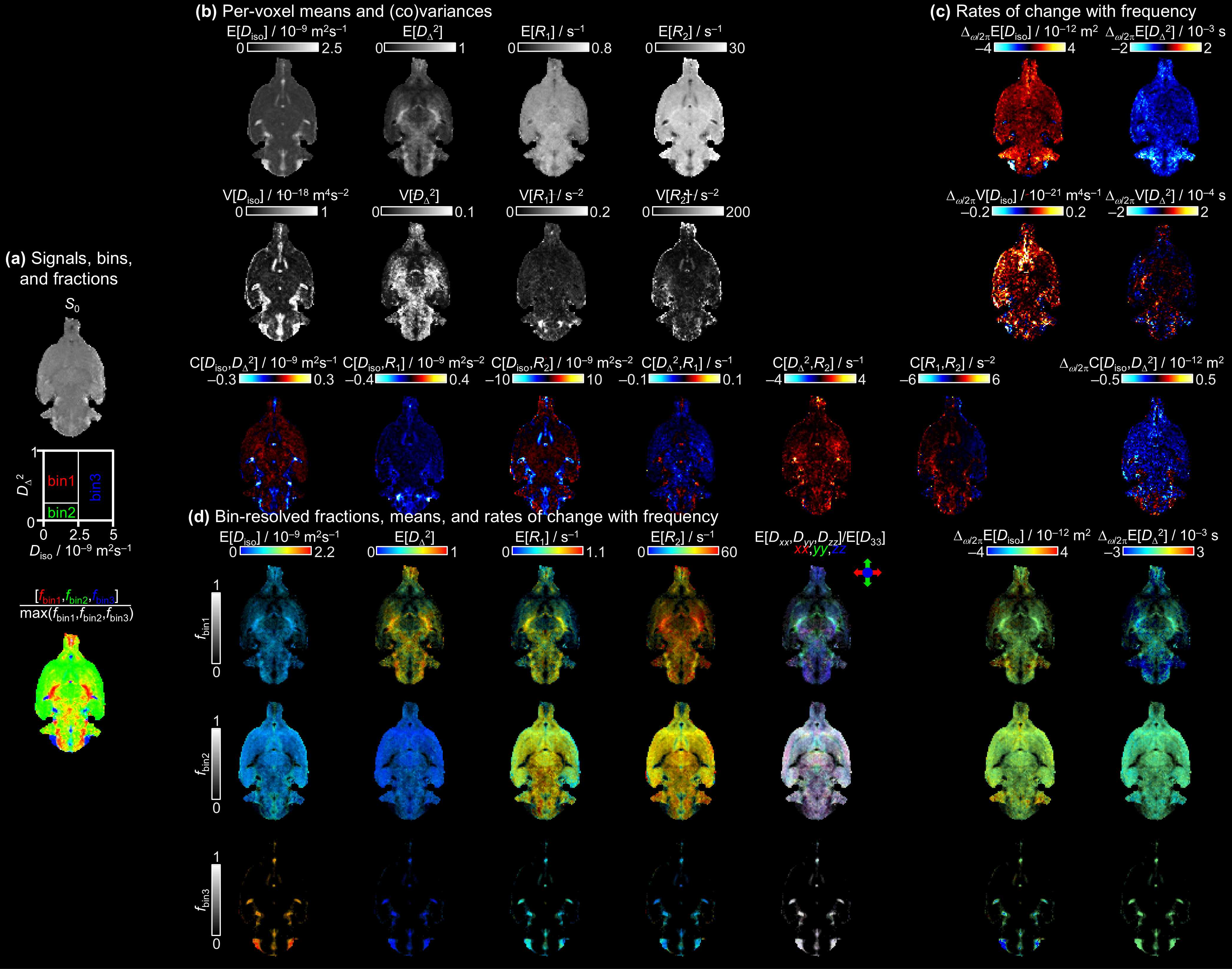


Supplementary Figure 4: Parameter maps derived from the per-voxel **D**(ω)-R1-R2-distributions of the in vivo rat brain slice 1.


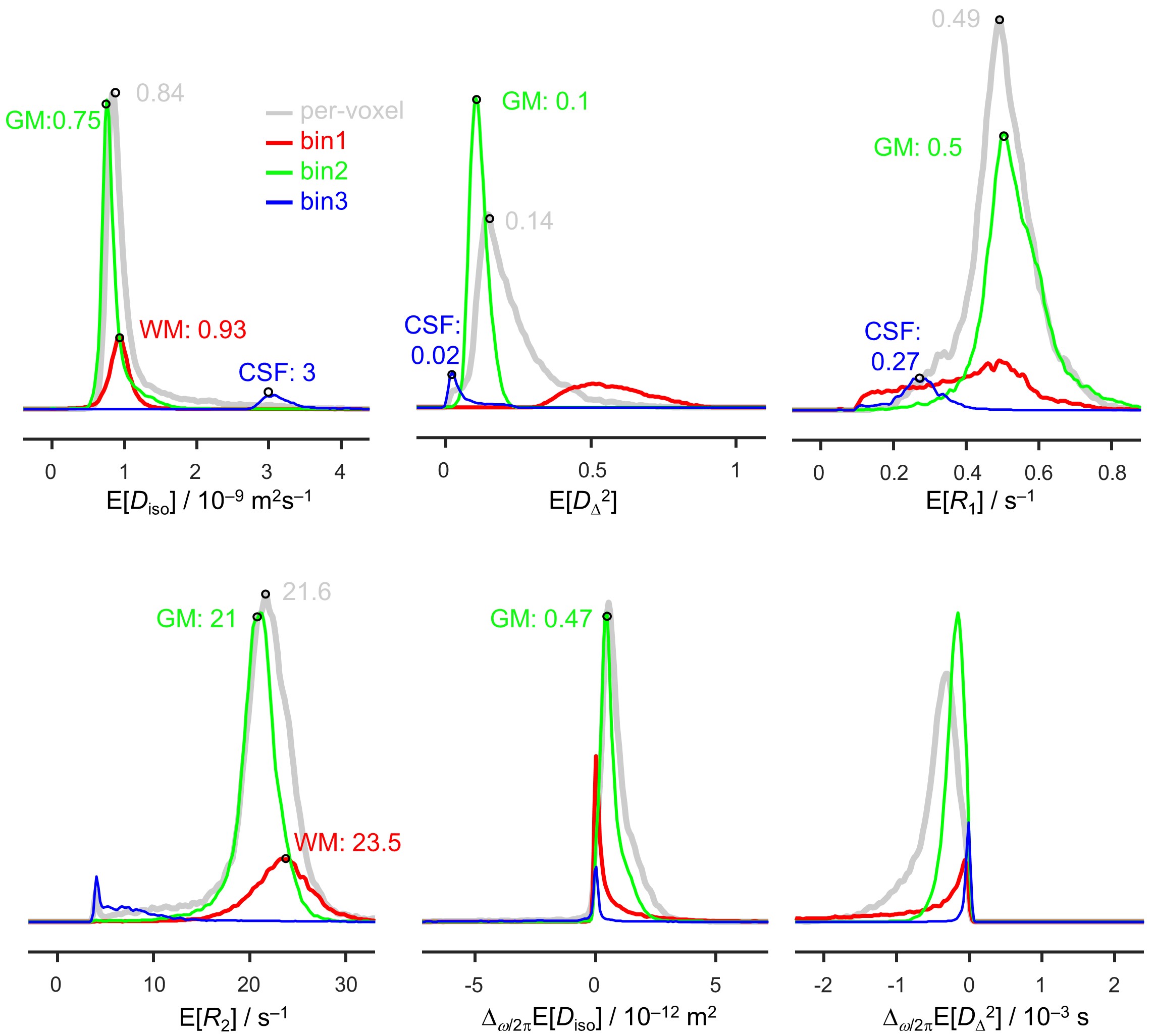


Supplementary Figure 5: Histograms of quantitative parameters median values across all voxels in the image. The histograms are obtained from the per-voxel (gray) and bin-resolved (red, green, and blue) maps of E[*D*_iso_], E[*D*_Δ_^2^], E[*R*_1_], E[*R*_2_], Δ*_ω_*_/2π_E[*D*_iso_], and Δ*_ω_*_/2π_E[*D*_Δ_^2^] in all slices and include weighting by *S*_0_, *f*_bin1_, *f*_bin2_, and *f*_bin3_. The abscissas cover the same ranges as the scale bars in Fig 3. Labeled points highlight representative values.


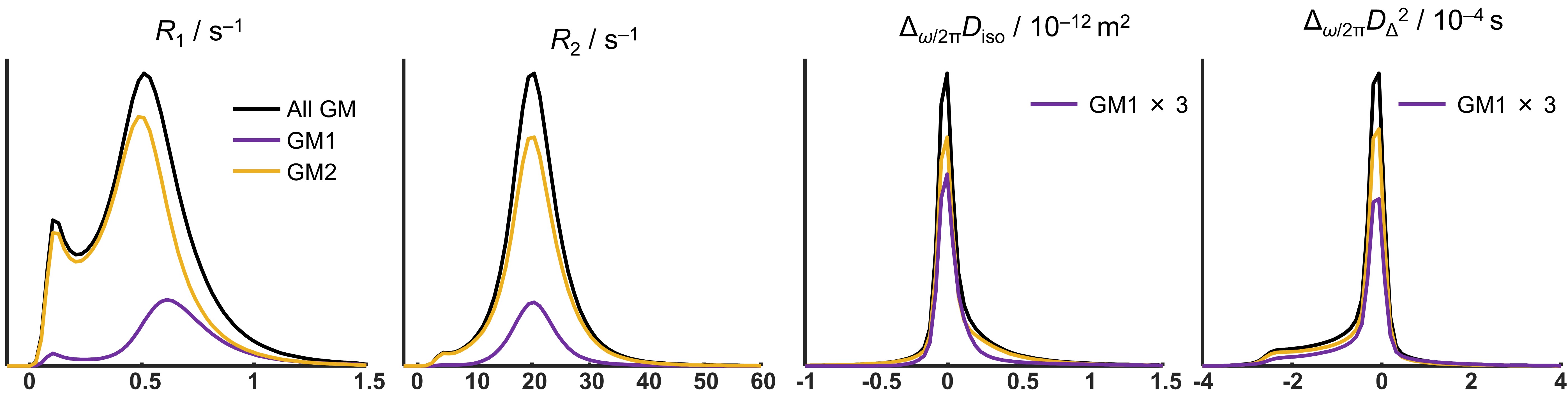


Supplementary Figure 6: R_1_, R_2_, Δ_ω/2π_D_iso_, and Δ_ω/2π_D_Δ_² raw distributions of bin 2 in black, GM1 in purple, and GM2 in yellow in the 5 rat brain slices. In the two right plots, the intensity of the GM1 distribution was multiplied by three to ease the comparison of the low-intensity non-zeros points.


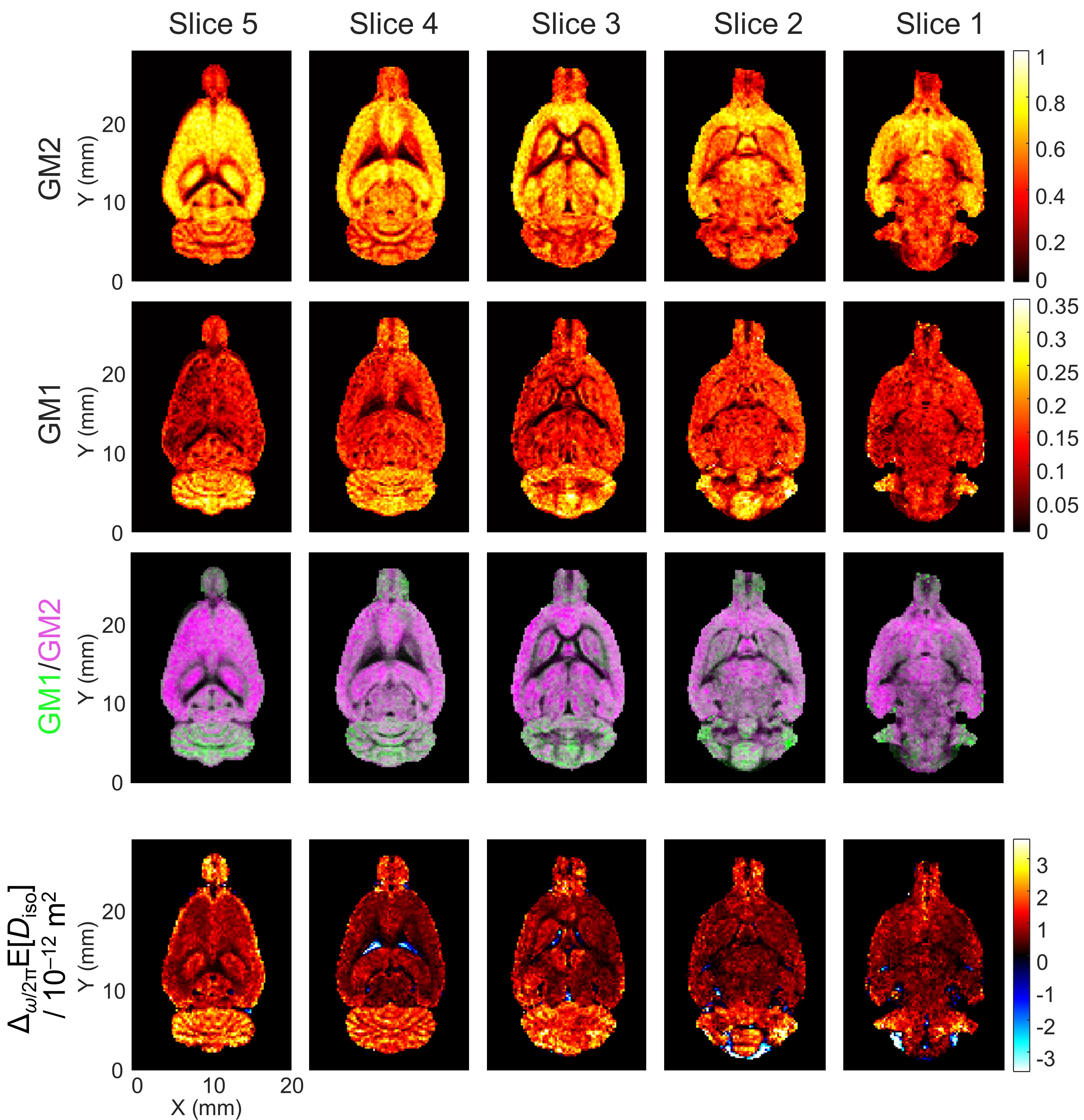


Supplementary Figure 7: parameter maps corresponding from top to bottom to: the S_0_ fraction of GM2, the S_0_ fraction of GM1, the overlap of GM1 over GM2 S_0_ fractions normalized by their maximum intensities in green and purple respectively, and the Δ_ω/2π_E[D_iso_] parameter maps reproduced here to ease comparison.
